# Stroke in chronic kidney disease

**DOI:** 10.4103/0971-4065.50672

**Published:** 2009-01

**Authors:** P. Rama Krishna, S. Naresh, G. S. R. Krishna, A. Y. Lakshmi, B. Vengamma, V. Siva Kumar

**Affiliations:** Department of Nephrology, Sri Venkateswara Institute of Medical Sciences, Tirupati, India; 1 Department of Radiology, Sri Venkateswara Institute of Medical Sciences, Tirupati, India; 2 Department of Neurology, Sri Venkateswara Institute of Medical Sciences, Tirupati, India

**Keywords:** Chronic kidney disease, stroke, hypertension, diabetes

## Abstract

Chronic kidney disease (CKD) is associated with a higher risk for stroke in studies from developed countries. This prospective study was conducted to study the clinical profile, management, and outcome of stroke in patients of chronic kidney disease who had been admitted in our institute during the period from December 2004 to December 2006. A higher incidence of stroke was found in men and in the fifth decade of life. Hypertension and diabetes were found in 88.8 and 48.1% of the patients respectively. CKD was detected for the first time during stroke evaluation in 55.5% of the patients. Stroke was due to cerebral infarction in 48.14% and due to cerebral hemorrhage in 40.7% of the patients. Surgical intervention was needed in 14.8% of all patients while stroke was managed medically in the rest. Over 70% of the patients were discharged after they showed improvement in the symptoms.

## Introduction

Chronic kidney disease is associated with a high risk for stroke.[1–4] Hypertension, diabetes mellitus, atherosclerosis, anemia, heparin usage, hyperlipidemias, hyperhomocystinemia, and protein malnutrition were cited as risk factors for stroke by several studies.[2–8] Stroke may manifest as infarction, hemorrhage, and sometimes, in a combination of these two. Infarcts were found to arise due to the involvement of carotid or vertibrobasilar arterial systems.[6] In hemorrhagic strokes, the bleed was found to be common in thalamic and basal ganglia regions.[4] Stroke in chronic dialysis patients is associated with high mortality.[3,4,6,8] This study was performed to ascertain the clinical profile, management, and the outcome of stroke in patients of chronic kidney disease at our center, a tertiary care referral center.

## Materials and Methods

All patients of stroke with chronic kidney disease admitted from December 2004 to December 2006 were the subjects of this study. The diagnosis of chronic kidney disease was made basing on the K/DOQI guide lines[1] and the staging of chronic kidney disease was based on the levels of serum creatinine (mild:1.5–3 mg/dL, moderate: 3–5 mg/dL, severe: >5 mg/dL). The diagnosis of stroke was made on the basis of the history, physical examination, and computed axial tomography of the head and brain. Magnetic resonance imaging (MRI) of the brain was done depending on the clinical need. Stroke was defined according to standard clinical and imaging criteria. The causes of stroke were further subdivided broadly into ischemic or hemorrhagic categories depending on the radiological appearance. Patients were carefully assessed for their risk factor status. Management was according to the standard protocol of the institution, and the outcome status was recorded.

## Results

A total of 1369 patients of chronic kidney disease were treated as inpatients from December 2004 to December 2006. Twenty-seven patients (1.97%) were found to have stroke. Male: female ratio was 19:8 and the age range of the patients varied from 32 to 80 years (mean age: 59.14 years). The types of chronic kidney disease observed were: diabetic nephropathy in 37%, hypertensive nephropathy in 18.5%, chronic glomerulonephritis in 11%, cystic kidney disease in 3.7%, and other types in 29% of the patients [Table 1]. Renal failure was mild in 25.95%, moderate in 37.03%, and severe in 37.03% of the patients. In 55.5% of the patients, chronic kidney disease was detected for the first time during their admission for stroke management.

**Table 1:** Type of underlying CKD in stroke patients

Disease	Incidence (%)
Diabetes mellitus	37
Hypertension	18.5
Chronic glomerulonephritis	11
Cystic diseases	3.7
Others	29

CKD: Chronic kidney disease

The stroke subtypes observed included infarction in 48%, hemorrhage in 40.7%, and both infarction and hemorrhage in 11.11% of the patients. The brain infarcts were found to arise due to large artery arteriosclerosis in 62.5% of the patients. Lacunar type infarcts were noted in 37.5% patients. The vascular territory of brain infarction includes: carotid system in 56.25% and the vertibrobasilar system in 43.75% of the patients. Stroke in the form of cerebral hemorrhage was detected in 40.74% of the patients. The distribution of the sites of hemorrhagic stroke were: thalamus in 38.46%, basal ganglion in 38.46%, subcortical in 15.38%, and cerebellum in 7.69% of the patients [Table 2]. The risk factors for stroke were: hypertension in 88.88% (uncontrolled hypertension 48.14%), anemia in 81.48%, diabetes in 48.14%, smoking in 33.33%, and hyperlipidemia in 14.81% of the patients [Table 3].

**Table 2:** Sites of hemorrhage

Site	Incidence (%)
Thalamus	38.46
Basal ganglia	38.46
Subcortical	15.38
Cerebellum	7.69

**Table 3:** Risk factor incidence

Risk factor	Incidence (%)
Hypertension	88.88
Anemia	81.48
Diabetes mellitus	48.14
Smokers	33.33
Hyperlipidemia	14.81

With regard to management of stroke, in addition to medical supportive management, craniotomy for evacuation of hematomas was performed in 14.8% of the patients. Hemodialysis support was needed in 18.5% of the patients for the management of renal failure. With regard to the outcome, 62.96% of the patients were discharged with improvement, 11.11% succumbed, 18.51% were discharged at their request, and 7.4% were in vegetative state at the time of discharge.

## Discussion

Chronic kidney disease (CKD) is associated with a high risk for stroke. The risk for stroke was found to be five times higher in CKD patients on dialysis in comparison to the general population.[2] In comparison to the general population, not only the stroke incidence, but also the death rate due to stroke were higher in the CKD and dialysis population.[2–6] In a study from Japan, stroke was found to be one of the leading causes of death accounting for 12.7% of total CKD-related deaths.[3]

The United States Renal Data System (USRDS) reported that the incidence of ischemic stroke was 5.3 times higher than that of hemorrhagic stroke in end stage renal disease. In comparison to the findings of earlier studies, we found the incidence of brain infarcts to be higher than that of brain hemorrhage. Inclusion of older patients with multiple risk factors for hemodialysis, usage of erythropoietin, and advanced diagnostic facilities in the form of magnetic resonance imaging (MRI) were some of the reasons reported for the increased incidence and recognition of brain infarcts in CKD patients. Improvements in dialysis membranes with the accompanying reduction in heparin usage were cited as the reasons for the observed decrease in the incidence of brain hemorrhage over time.[5,6]

The increased prevalence of large artery disease, vertibrobasilar steal with the use of forearm AV access during hemodialysis, decrease in intravascular blood volume secondary to hemodialysis, autonomic neuropathy, and hyperhomocystinemia were cited as important factors for the higher incidence of brain infarcts in CKD and dialysis patients.[6] The increased incidence of cerebral hemorrhage in CKD and dialysis patients was ascribed to the high prevalence of hypertension, protein malnutrition, and hypoalbuminemia, which directly affected erythrocyte deformability and endothelial dysfunction.[7,8] Cerebral hemorrhage was often observed in the thalamic and basal ganglion regions.[4] Brain infarcts were frequently found to occur during or shortly after dialysis procedures, whereas cerebral hemorrhage was usually found to occur 35.5 hours after the last dialysis.[7]

Elevated serum creatinine by itself was found to be a strong and independent predictor of the increase in risk and the outcome after stroke. It has been suggested that serum creatinine is a marker for generalized vascular disease.[9,10]

In this study, about 2% of the CKD patients were found to have stroke, with men outnumbering women. Stroke was secondary to infarcts in 48% and due to hemorrhage in 41% patients, while the rest had both infarcts and hemorrhage. In CKD patients with stroke, the severity of renal failure was mild in 26%, moderate in 37%, and severe in 37%. Our observations concur with reported literature that brain infarcts are more common than brain hemorrhage, infarcts being mainly due to large artery arteriosclerosis and hemorrhage most commonly located in the thalamic and basal ganglion regions.[4,6]

Hypertension, anemia, diabetes, smoking, and hyperlipidemia were the important risk factors in the risk factor analysis. Interestingly, CKD was detected for the first time during admission for stroke management in over 50% of the patients. In patients with infarcts, the lesions were found to be due to large artery arteriosclerosis in ten patients and due to lacunar infarcts in six patients.

## Conclusions

In our study, the incidence of stroke presentation was 1.97%. Out of 1369 CKD patients studied in the two-year period from December 2004 to December 2006, stroke was found to be more frequent in males and in the middle age groups. Hypertension, anemia, and diabetes were found to be important risk factors for stroke.[5,6] Brain infarcts were more prevalent than brain hemorrhage. Active management improved the outcome in 62.9% of the patients. All patients who succumbed had hemorrhage, an observation that highlights the sinister significance of hemorrhage in CKD patients.[3] Therefore, clinicians should have high index of suspicion for CKD in stroke patients.
